# An anti-eCIRP strategy for necrotizing enterocolitis

**DOI:** 10.1186/s10020-024-00935-3

**Published:** 2024-09-20

**Authors:** Colleen P. Nofi, Jose M. Prince, Mariana R. Brewer, Monowar Aziz, Ping Wang

**Affiliations:** 1 Center for Immunology and Inflammation, the Feinstein Institutes for Medical Research, 350 Community Dr., Manhasset, NY 11030 USA; 2 Elmezzi Graduate School of Molecular Medicine, Manhasset, NY 11030 USA; 3 Department of Surgery, Zucker School of Medicine, Manhasset, NY 11030 USA; 4 Department of Pediatrics, Zucker School of Medicine, Manhasset, NY 11030 USA; 5 Department of Molecular Medicine, Zucker School of Medicine, Manhasset, NY 11030 USA

**Keywords:** eCIRP, MFG-E8, MOP3, Necrotizing enterocolitis, Inflammation

## Abstract

**Background:**

Necrotizing enterocolitis (NEC) is a severe gastrointestinal disease characterized by intestinal inflammation and injury, with high mortality risk. Extracellular cold-inducible RNA-binding protein (eCIRP) is a recently discovered damage-associated molecular pattern that propagates inflammation and tissue injury; however, the role of eCIRP in NEC remains unknown. We hypothesize that eCIRP exacerbates NEC pathogenesis and the novel eCIRP-scavenging peptide, milk fat globule-epidermal growth factor-factor VIII (MFG-E8)-derived oligopeptide 3 (MOP3), attenuates NEC severity, serving as a new therapeutic strategy to treat NEC.

**Methods:**

Stool samples from premature neonates were collected prospectively and eCIRP levels were measured. Wild-type (WT) and CIRP^−/−^ mouse pups were subjected to NEC utilizing a combination of hypoxia and hypercaloric formula orogastric gavage with lipopolysaccharide supplementation. In parallel, WT pups were treated with MOP3 or vehicle. Endpoints including NEC severity, intestinal injury, barrier dysfunction, lung injury, and overall survival were determined.

**Results:**

Stool samples from NEC neonates had elevated eCIRP levels compared to healthy age-matched controls (p < 0.05). CIRP^−/−^ pups were significantly protected from NEC severity, intestinal injury, bowel inflammation, intestinal barrier dysfunction, lung injury, and systemic inflammation. NEC survival was 100% for CIRP^−/−^ pups compared to 65% for WT (p < 0.05). MOP3 treatment recapitulated the benefits afforded by CIRP-knockdown, preventing NEC severity, improving inflammatory profiles, and attenuating organ injury. MOP3 treatment improved NEC survival to 80% compared to 50% for vehicle treatment (p < 0.05).

**Conclusions:**

eCIRP exacerbates NEC evidenced by protection with CIRP-deficiency and administration of MOP3, a CIRP-directed therapeutic, in a murine model. Thus, eCIRP is a novel target with human relevance, and MOP3 is a promising treatment for lethal NEC.

**Supplementary Information:**

The online version contains supplementary material available at 10.1186/s10020-024-00935-3.

## Introduction

Necrotizing enterocolitis (NEC) is a devastating disease predominantly afflicting premature infants (Neu and Walker 2011). Owing to its elusive pathophysiology, limited treatment options in NEC have resulted in high morbidity and mortality, with mortality risk as high as 50% in those undergoing surgery (Bazacliu and Neu 2019). Clinically, the disease is characterized by bowel inflammation and ischemia that progresses to necrosis and perforation (Neu and Walker 2011). The development of NEC is complex and driven by poorly characterized pathways, orchestrated in part by bacterial colonization, increased expression of toll-like receptors, tight-junction impairment, decreased absorption, dysmotility, and impaired perfusion of the gut (Cho et al. 2020; Niño et al. 2016; Aziz et al. 2022). Despite advances in the discovery of pathogenic underpinnings of NEC, critical gaps have stalled the successful transition of scientific discovery to impact clinical care in improving outcomes.

The role of damage-associated molecular patterns (DAMPs), and more specifically of chromatin-associated molecular patterns (CAMPs) in gut inflammatory disorders has been previously demonstrated in premature infants (Nofi et al. 2022). Recently, extracellular cold-inducible RNA-binding protein (eCIRP) was identified as a novel CAMP found to propagate inflammation and worsen tissue injury (Qiang et al. 2013). High levels of eCIRP have been directly correlated with inflammatory conditions, including increased sepsis severity in neonates (Denning et al. 2020). Furthermore, targeting eCIRP has been shown to reduce organ dysfunction in preclinical models of sepsis (Denning et al. 2020); however, eCIRP’s role in worsening outcomes and as a target for therapeutic intervention in NEC has not been previously investigated.

Given the detrimental impact of eCIRP in propagating the inflammatory cascade, we recently investigated mechanisms of targeting eCIRP and identified a new protein–protein interaction whereby milk-fat globule epidermal growth factor-factor VIII (MFG-E8) scavenges eCIRP to confer an anti-inflammatory impact (Nofi et al. 2024a). MFG-E8 is a secretory anti-inflammatory glycoprotein ubiquitously expressed in immune reactive cells and is highly expressed in the lactating mammary gland (Aziz et al. 2011). Based on eCIRP-MFG-E8 interaction, we developed a novel peptide, MFG-E8-derived oligopeptide 3 or MOP3 that replicates MFG-E8’s function to scavenge eCIRP and successfully improved outcomes in experimental inflammatory diseases (Nofi et al. 2024a, 2024b).

Although the role of CAMPs in exaggerating inflammatory pathways has long been understood, limited attention has been made to the impact of eCIRP in causing immune dysregulation and contributing to the progression of NEC. Thus, this work aimed to uncover the role of eCIRP in worsening NEC pathogenesis and elucidate the therapeutic potential of an eCIRP scavenger, MOP3, designed to clear eCIRP and attenuate NEC severity.

## Methods

### Human samples

Institutional review board approval (Northwell Health IRB #19-0981) was obtained to prospectively collect stool samples from neonates admitted to the neonatal intensive care unit (NICU) at a tertiary children’s hospital. The medical record was reviewed for clinical information, allowing for identification of neonates who developed NEC. Stool samples from NEC neonates and controls age-matched by gestational age at birth (who were not diagnosed with NEC or sepsis during NICU admission) were collected, aliquoted, and frozen at −80 °C. At the time of analysis, ~ 50 mg of stool were homogenized in PBS and the supernatant was tested for eCIRP levels with a human enzyme-linked immunosorbent assay (ELISA) kit according to the manufacturer’s instructions (Cusabio Biotech Co., Houston, TX).

### Experimental animals

House-bred C57BL/6 wild-type (WT) and eCIRP^−/−^ age-matched male and female mice were maintained in a temperature-controlled room under 12-h light/dark cycles and fed a standard rodent diet. For all animal experiments, mouse pups utilized were 5–7 days old and ranged from 3–4 g in body weight (BW). Mouse pups were utilized regardless of sex, as it cannot be readily determined by external features at this age and weight. Animal experiments were conducted in strict compliance with the National Institutes of Health Guidelines for the Care and Use of Laboratory Animals, and the study procedures were approved by the Feinstein Institutes for Medical Research Institutional Animal Care and Use Committee (IACUC).

### Murine model of necrotizing enterocolitis

Experimental NEC was induced in wild-type (WT) and CIRP^−/−^ pups (age 5–7 days old) as previously described (Zani et al. 2008, 2016; Good et al. 2015; Leaphart et al. 2007; Nolan et al. 2021). Briefly, pups were separated from mothers on the first day of the model and housed at 30 °C (ThermoCare Portable Animal Intensive Care Unit). Pups were orally gavage-fed hypercaloric formula [Similac Advance (Abbott Nutrition):Esbilac (canine milk replacer, PetAg); 2:1] with a 24-Gauge angiocatheter. Gavage feeds were performed 5 times/day consisting of 50 μl/g of mouse body weight. On days 1 and 2 of the NEC model, the third feed was supplemented with LPS (lipopolysaccharide from *Escherichia coli 055:B5,* Sigma-Aldrich Company*;* 4 μg/g/day) as previously described (Zani et al. 2008, 2016). Pups were subjected to hypoxia for 10 min 2 times/day (5% O_2_-95% N_2_, Modular Incubator Chamber, Embrient Inc) verified by monitoring with an O_2_ gas detector (BW O_2_ Gas Alert Clip), for a total of 4 days. In a treatment model, mice were randomized and treated with either MOP3 (*i.p.*, 20 μg/g body weight daily) or volume-equivalent vehicle. Control (i.e., non-NEC, sham) animals remained with their mothers and were breastfed ad libitum. Samples were harvested at the end of the model (Leaphart et al. 2007). Pups with mortality prior to experimental endpoints were excluded from tissue and serum analyses. For survival studies, NEC-induced mortalities were observed as countable events. Only mice that had obvious non-NEC mortality (i.e. gavage of formula into the chest or hemorrhage from injection) were excluded.

### NEC severity grading

Ileum resections were fixed in 10% formalin, embedded in paraffin, and sectioned (5 µm) for hematoxylin and eosin (H&E) staining. NEC severity was assessed through blinded evaluation using previously validated scoring system based on degree of injury as follows: 0, no damage; 1, slight submucosal and/or lamina propria separation; 2, moderate separation of submucosa and/or lamina propria and/or edema in submucosal and muscular layers; 3, severe separation of submucosa and/or lamina propria and/or severe edema in submucosa and muscular layers, regional villous sloughing; and 4, necrosis and destruction of the intestinal epithelium (Sodhi et al. 2018). Two images were taken per sample and NEC severity was averaged across images.

### Intestinal permeability assay

Intestinal permeability experiments were performed using oral gavage of FITC-4 kDa dextran (FD4 28 A, Sigma Aldrich, St Louis, MO) with assessment of fluorescence in the serum as a marker for intestinal barrier dysfunction, as previously described (Oami and Coopersmith 2021). Pups received oral gavage of 100 μL of FD4 at 22 mg/mL via 24-gauge angiocath 3 h prior to collection of blood. The supernatant-containing serum was separated and analyzed for FD4 using excitation wavelength of 485 and emission wavelength of 528 nm. Relative fluorescence intensity was measured by expressing fluorescence measurements as fold-change from sham control.

### Synthesis and administration of MOP3

MOP3, an opsonic therapeutic peptide was developed as previously described (Nofi et al. 2024a). Briefly, the 18-amino acid (AA) sequence of MOP3 was derived through synthesis of 15-AA from MFG-E8 discoidin domain 2 (designed to bind eCIRP) with an additional 3-AA tail, Arg-Gly-Asp (RGD, designed to promote phagocytosis and clearance). The 18-AA peptide was synthesized from GenScript USA Inc (Piscataway, NJ) with 96.6% purity. MOP3 was solubilized to a concentration of 10 mg/mL in DMSO and then diluted to 2 mg/mL in normal saline. MOP3 was stored as a lyophilized powder at −20 °C and freshly solubilized for each in vivo experiment.

### Measurement of intestinal length

At the time of collection, a laparotomy was performed, and the enteric tract was eviscerated. The length of the small bowel (from pylorus to ileocecal juncture) was determined by using a ruler and allowing the intestine to lay straight without tension.

### Terminal deoxynucleotidyl transferase dUTP nick end labeling assay

Terminal deoxynucleotidyl transferase dUTP nick end labeling assay (TUNEL) staining was performed on 5 µm small bowel and lung sections using a commercially available fluorescence In Situ Cell Death Detection Kit (Roche Diagnostics), according to the manufacturer's instructions. The nuclei were counterstained with DAPI (Vectashield Antifade Mounting Media; H-2000). Three representative images were taken for each sample, analyzed for TUNEL ( +) cells using ImageJ, Fiji software (version 2.1.051) and averaged across representative images (Schindelin et al. 2012).

### Zona occludens-1 immunofluorescence staining

The tight junctional protein, zonula occludens-1 (ZO-1) was assessed in the small intestine by immunofluorescent staining. Paraffin-embedded sections of ileum were dewaxed in xylene, rehydrated in ethanol, and heated at 95 °C for 10 min in 0.92% citric acid buffer (Vector Laboratories, Burlingame, CA). After cooling, slides were washed and blocked in 10% normal donkey serum with 1% BSA for 2 h. The primary antibody against ZO-1 (Abcam, Waltham MA; catalog no: ab96587) was used to incubate intestinal tissue sections overnight at 4 °C. After washing with 0.025% Triton X-100, the secondary, Alexa Fluor 488-conjugated antibody (Inivtrogen, Carlsbad, CA; catalog no: A-21206) was used for a 1-h incubation at room temperature. Slides were then copiously washed and counterstained for nuclei with 4',6-diamidino-2-phenylindole (DAPI, Vectashield Antifade Mounting Media, H-2000).

### Enzyme-linked immunosorbent assay and organ injury markers

Systemic levels of IL-6 and TNFα were measured by ELISA kits (BD Biosciences, San Diego, CA), and eCIRP levels were quantified with mouse CIRP-specific ELISA kits (American Research Products, Waltham, MA) according to the manufacturers’ instructions. Organ injury marker lactate dehydrogenase (LDH) was measured in fresh supernatant-containing serum using a commercial calorimetric assay kit (Pointe Scientific, Canton, MI) by the manufacturer’s instructions.

### Lung histopathology

Lungs were fixed in 10% formalin, embedded in paraffin, and sectioned (5 µm) for H&E staining. Sections were analyzed under light microscopy and 3 images were captured per lung at × 200 magnification. Images were the blindly scored and averaged for the degree of lung injury using the system previously validated by the American Thoracic Society (Matute-Bello et al. 2011). This score is based on the presence of proteinaceous debris in the airspaces, degree of septal thickening, and neutrophil infiltration in the alveolar and interstitial spaces.

### Real-time polymerase chain reaction

RNA was isolated from tissue using Illustra RNAspin Mini RNA Isolation kit (GE Healthcare, Chicago, IL). Briefly, tissues were homogenized using β-mercaptoethanol and lysis solution, and total tissue RNA was extracted according to the manufacturer’s instructions. RNA was then reverse-transcribed into complementary deoxyribonucleic acid (cDNA) using purified RNA and the RT^2^ First Strand Kit. A Step One Plus real-time PCR machine (Applied Biosystems, Thermo Fisher Scientific) was used for amplification and analyses. CT values were exported, and data analyses were performed at QIAGEN’s GeneGlobe Data Analysis Center. Forward and reverse primer sequences for respective targets are provided in Supplemental Table 1.

### Statistical analysis

Data represented in the figures are expressed as mean ± SEM. For comparison among multiple groups, one-way ANOVA was used for normally distributed data. Significant differences between groups were determined by Tukey’s method. Survival rates were analyzed by the Kaplan–Meier estimator and compared using a log-rank test. Significance was considered between study groups as *p* ≤ 0.05. Data analyses were carried out using GraphPad Prism graphing and statistical software (GraphPad Software, version 10.0.2).

## Results

### eCIRP exacerbates NEC pathogenesis and intestinal injury

**Fig. 1:**
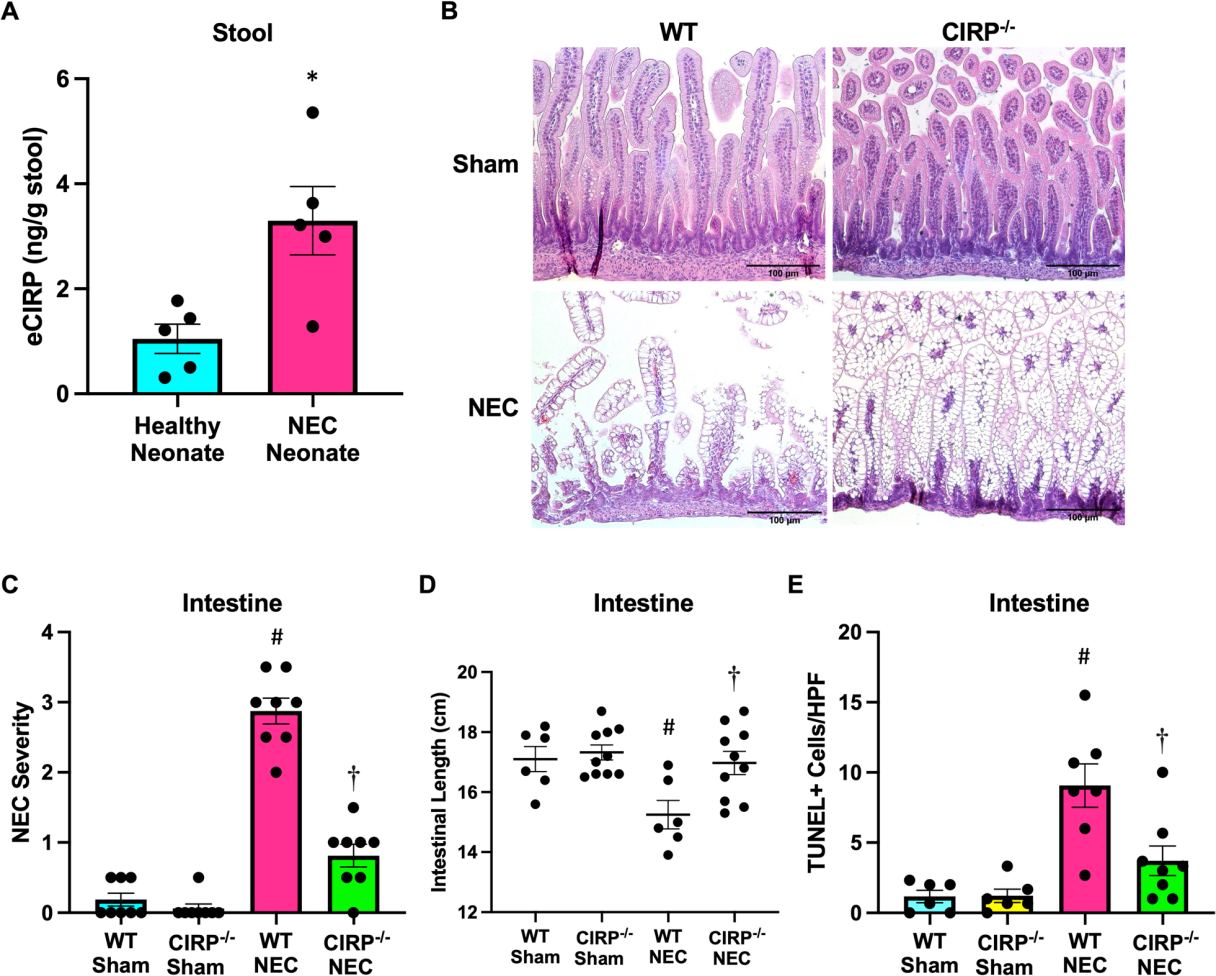
eCIRP exacerbates NEC pathogenesis and intestinal injury. **A** eCIRP levels detected in the stool of neonates with NEC and age-matched control neonates without NEC. **B** NEC was induced in WT and CIRP^−/−^ pups and intestines were collected after 4 days of NEC. Age- and litter-matched WT and CIRP^−/−^ sham counterpart intestines were collected for comparison. Representative H&E images of sham and NEC intestines. Original magnification: 200x, Scale bar: 100 μm. **C** Quantification of NEC severity scoring of H&E intestinal sections. **D** Quantification of intestinal length from sham and NEC intestines. **E** Quantification of terminal deoxynucleotidyl transferase dUTP nick end labeling (TUNEL)–positive cells from intestinal sections. All experiments were performed 3 times, and all quantitative data were used for analysis (N = 6–10). Data are expressed as mean ± SEM and compared by One-way ANOVA and Tukey’s method. *p < 0.05 v. healthy neonate, ^#^p < 0.05 vs. sham, ^†^p < 0.05 vs. WT NEC

Neonates who were diagnosed with NEC ranged in gestational age from 24 to 27 weeks and were diagnosed at a median age of 21 days. Additional clinical information is provided in Supplemental Table 2. Stool from NEC neonates exhibited significantly elevated levels of eCIRP by 3.1-fold compared to stool from healthy, non-NEC gestational age-matched neonates (Fig. 1A). Further, in our murine model, induction of NEC was associated with a 64-fold increase in systemic eCIRP levels compared to WT sham pups. WT NEC mice demonstrated increased NEC severity scores in ileal sections compared to WT sham mice, whereas NEC severity was reduced in CIRP^−/−^ pup NEC intestines (Fig. 1B, C). This induction of NEC resulted in progressive loss of bowel length in respective mice, whereas there was preservation of bowel length observed in CIRP^−/−^ mice compared to WT in NEC (Fig. 1D). To investigate intestinal injury further, TUNEL staining was performed, revealing increased number of apoptotic cells in NEC intestines compared to sham and less NEC-induced apoptotic intestinal cells in CIRP^−/−^ mice compared to WT (Fig. 1E). Thus, eCIRP significantly contributes to NEC pathogenesis and intestinal injury.

### eCIRP deficiency protects against intestinal inflammation and barrier dysfunction

**Fig. 2:**
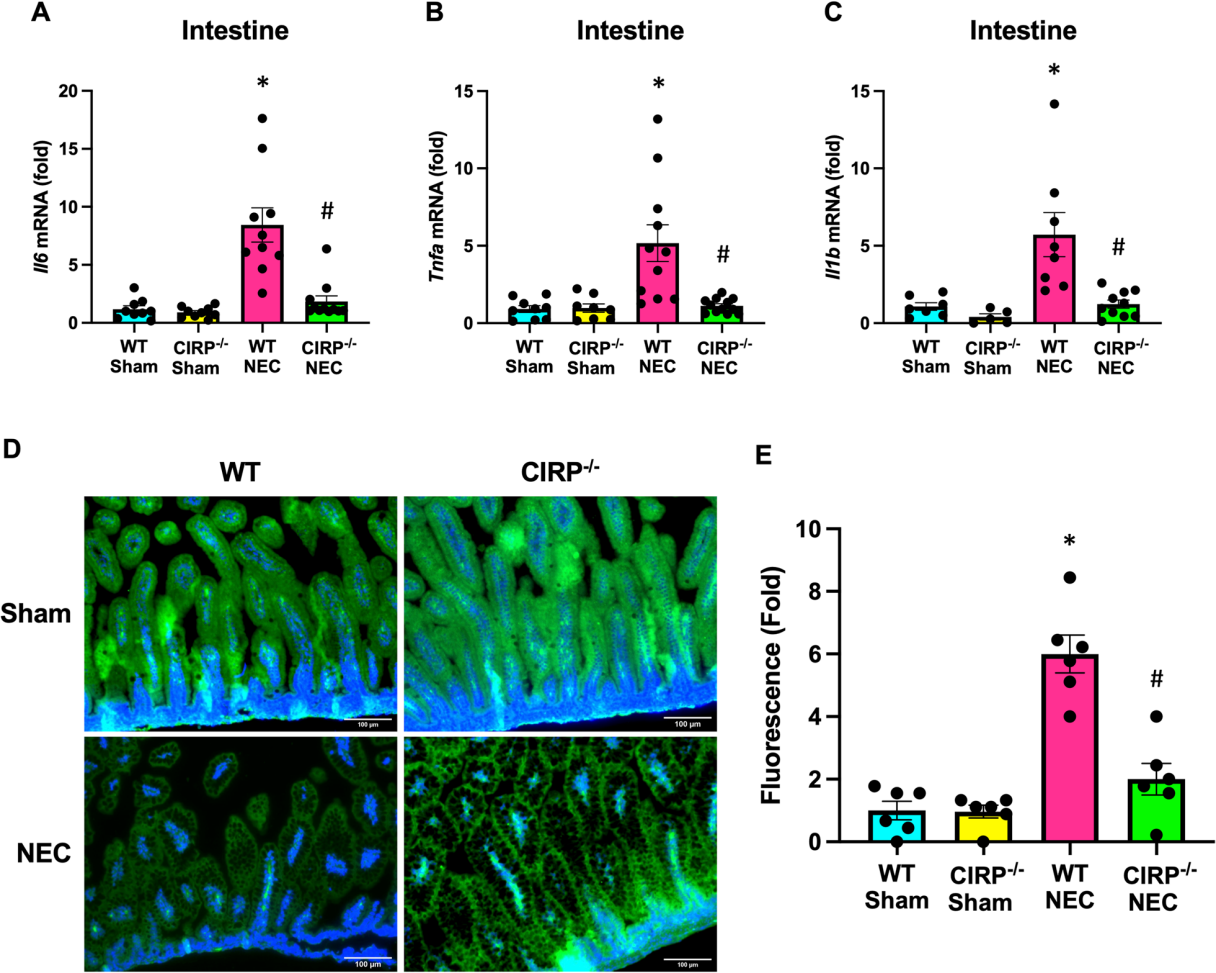
eCIRP deficiency protects against intestinal inflammation and barrier dysfunction. NEC was induced in WT and CIRP^−/−^ pups and blood and intestines were collected after 4 days of NEC. Age and litter-matched WT and CIRP^−/−^ sham counterpart blood and intestines were collected for comparison. **A**–**C** Intestines were frozen and mRNA expression of IL-6, TNFα, and IL-1β was measured by PCR. **D** Sectioned intestines were stained by immunofluorescence for ZO-1 (green) and nuclear counterstained (blue). Original magnification: 200x, Scale bar: 100 μm. **E** Pups were administered FITC-dextran (FD4) by orogastric gavage 3-h prior to collection of blood and serum was analyzed for fluorescence intensity (expressed as fold-change from WT sham) as a measure of intestinal permeability. All experiments were performed 3 times, and all quantitative data were used for analysis (N = 6–12). Data are expressed as mean ± SEM and compared by One-way ANOVA and Tukey’s method. *p < 0.05 vs. WT sham, ^#^p < 0.05 vs. WT NEC

To investigate the link between induction of NEC and intestinal inflammation, whole intestines were analyzed for levels of pro-inflammatory cytokines. WT NEC pups exhibited increased intestinal mRNA levels of IL-6, TNF-α, and IL-1β, whereas CIRP^−/−^ NEC intestines had significantly reduced levels compared to WT, and similar levels to those observed in healthy sham pups (Fig. 2A–C).

Local intestinal injury and inflammation may progress to alteration of the intestinal barrier and further contribute to ongoing insult. Thus, we sought to evaluate tight-junctional protein, zona occludens-1 (ZO-1), which plays an important a role in maintaining the intestinal barrier (Moore et al. 2016). Upon induction of NEC in WT mice, we observed depletion of ZO-1 expression in the intestinal epithelium by green immunofluorescence. Conversely, ZO-1 depletion in NEC was blunted in CIRP^−/−^ NEC pup intestines (Fig. 2D). Finally, to probe the functional effect of CIRP-knockdown on preserving the intestinal barrier in NEC, a functional experiment was performed using FD4 enteric gavage and evaluating for intestinal translocation of FD4 extraluminally. Upon measurement of sera fluorescence, WT NEC pups had increased relative fluorescence compared to sham, whereas serum from CIRP^−/−^ NEC pups had significantly lower fluorescence, indicating an intact intestinal barrier (Fig. 2E). These data support that eCIRP contributes to intestinal inflammation and leaky intestinal barrier in NEC.

### eCIRP worsens NEC-induced acute lung injury

**Fig. 3:**
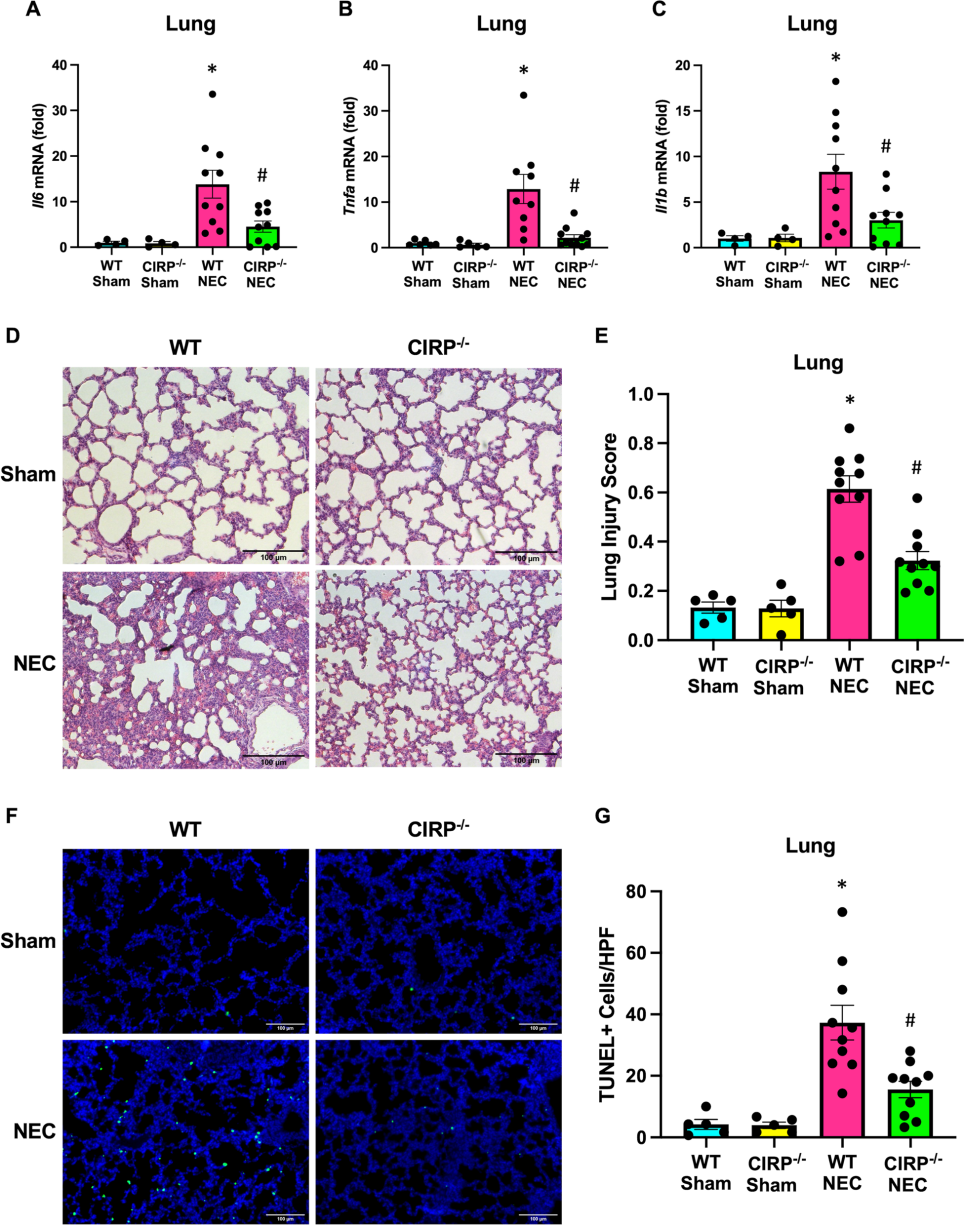
eCIRP worsens NEC-induced acute lung injury. NEC was induced in WT and CIRP^−/−^ pups and lungs were collected after 4 days of NEC. Age and litter-matched WT and CIRP^−/−^ sham counterpart lungs were collected for comparison. **A**–**C** Whole lung tissue was frozen and mRNA expression of IL-6, TNFα, and IL-1β were measured by PCR. **D** Representative H&E images of NEC and sham lungs. Original magnification: 200x, Scale bar: 100 μm. **E** Quantification of lung injury of representative H&E lung sections by validated lung injury scoring. **F** Representative terminal deoxynucleotidyl transferase dUTP nick end labeling assay (TUNEL) staining (green) of sham and NEC lungs for apoptotic cells with nuclear counterstain (blue). Original magnification: 200x, Scale bar: 100 μm. **G** Quantification of TUNEL–positive cells from representative lung sections. All experiments were performed 3 times, and all quantitative data were used for analysis (N = 6–11). Data are expressed as mean ± SEM and compared by One-way ANOVA and Tukey’s method. *p < 0.05 vs. WT sham, ^#^p < 0.05 vs. WT NEC

One of the most significant and long-term sequelae of NEC is the development of severe inflammatory lung disease (Jia et al. 2016). Thus, we sought to examine the impact of CIRP-knockdown on NEC-associated lung injury. mRNA levels of inflammatory cytokines, IL-6, TNF-α, and IL-1β were increased in the lungs of WT pups subjected to NEC, whereas these levels were significantly reduced in CIRP^−/−^ counterpart lungs (Fig. 3A–C). Lung injury was further assessed by histopathologic analysis of H&E lung sections. When blindly scored using the American Thoracic Society scoring system (Matute-Bello et al. 2011), WT NEC pup lungs were found to have significantly greater degree of lung injury compared to sham, whereas NEC-induced lung injury was prevented in CIRP^−/−^ pup lungs (Fig. 3D, E). Finally, degree of cellular apoptosis in the lung was assessed using TUNEL staining. There was a significant degree of cellular apoptosis in the lungs of WT pups subjected to NEC which was attenuated in the lungs of CIRP^−/−^ mice (Fig. 3F, G). Collectively, these data show eCIRP contributes to lung inflammation and injury in NEC.

### eCIRP deficiency protects against systemic inflammation and mortality in NEC

**Fig. 4:**
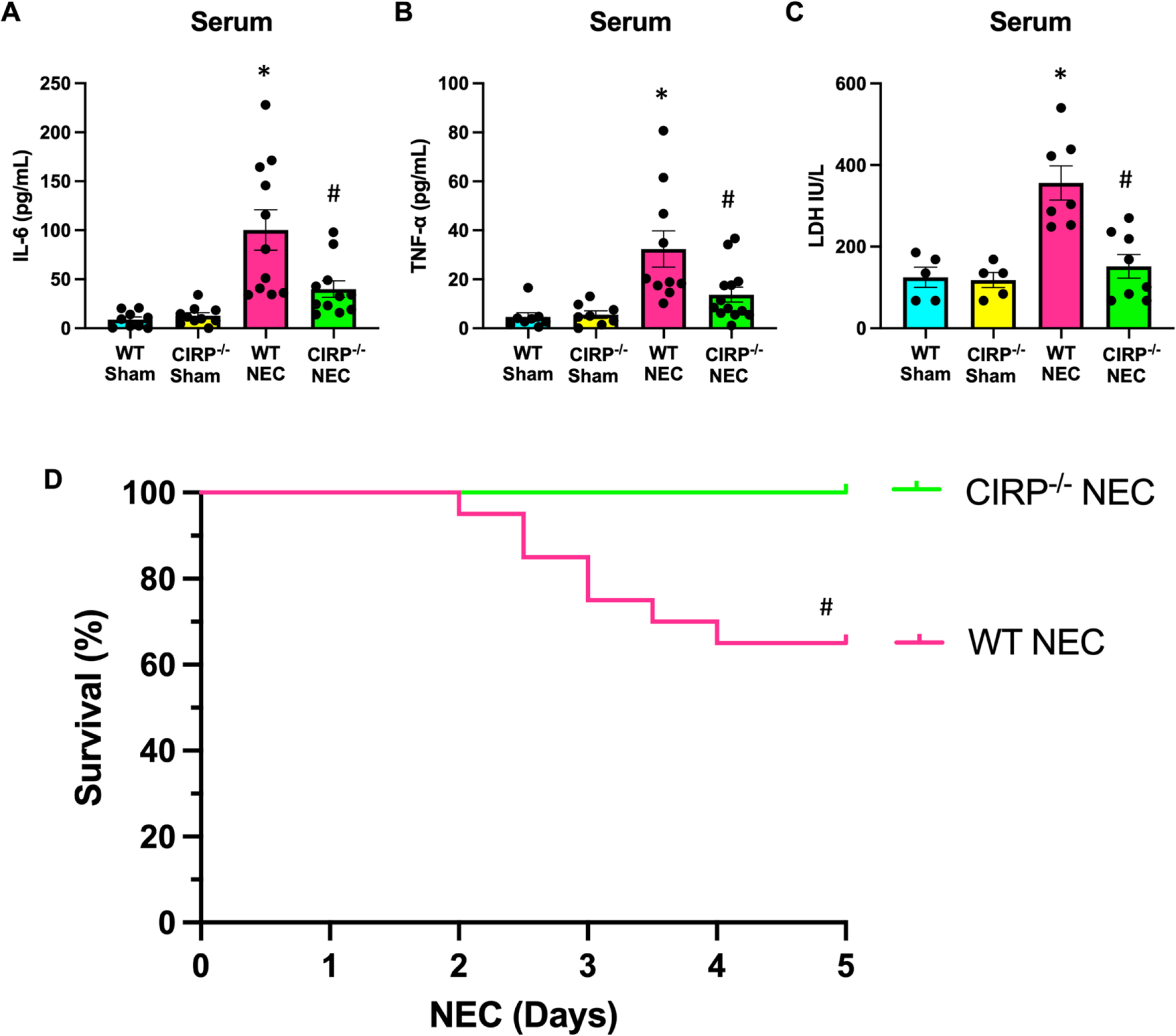
eCIRP deficiency protects against systemic inflammation and mortality in NEC. NEC was induced in WT and CIRP^−/−^ pups and blood was collected after 4 days of NEC. Age and litter-matched WT and CIRP^−/−^ sham counterpart blood was collected for comparison. **A**–**B** Serum was separated and analyzed for systemic IL-6 and TNFα levels by ELISA. **C** Serum was analyzed for LDH levels by calorimetric assays. All experiments were performed 3 times, and all quantitative data were used for analysis (N = 6–13). Data are expressed as mean ± SEM and compared by One-way ANOVA and Tukey’s method. **D** WT and CIRP^−/−^ pups were subjected to NEC for 4 days and monitored for differences in mortality in a 5-day survival study. Overall survival at 5-days was 100% for CIRP^−/−^ pups compared to 65% for WT pups (p = 0.004 by Kaplan Meier and log-rank test, N = 20/group). *p < 0.05 vs. WT sham, ^#^p < 0.05 vs. WT NEC; For survival curve: #p < 0.05 vs. CIRP^−/−^ NEC

In NEC, the inflammatory response is not limited to the intestine, but impacts the entire body causing widespread inflammatory damage (Moore et al. 2016). Expectedly in our murine model, systemic levels of IL-6, TNF-α, and LDH were all elevated in WT NEC pups. Comparatively, these levels were significantly attenuated in CIRP^−/−^ NEC pups (Fig. 4A–C). Ultimately, the feared outcome of NEC occurs when the uncontrollable inflammatory state overwhelms end-organ function, resulting in death. To evaluate if improved inflammatory profiles translated to differences in mortality, a 5-day survival study was performed with WT and CIRP^−/−^ NEC pups. At 5-days, there was a 35% mortality rate for WT NEC pups. Remarkably, in the same model and under the same conditions, there were no mortalities observed in CIRP^−/−^ NEC pups (Fig. 4D). Together, these data strongly suggest that eCIRP plays a critical role in exacerbating NEC, worsening the associated inflammatory response, and contributing to lethality in this disease.

### MOP3 protects against NEC pathogenesis, intestinal injury and inflammation, and barrier dysfunction

**Fig. 5:**
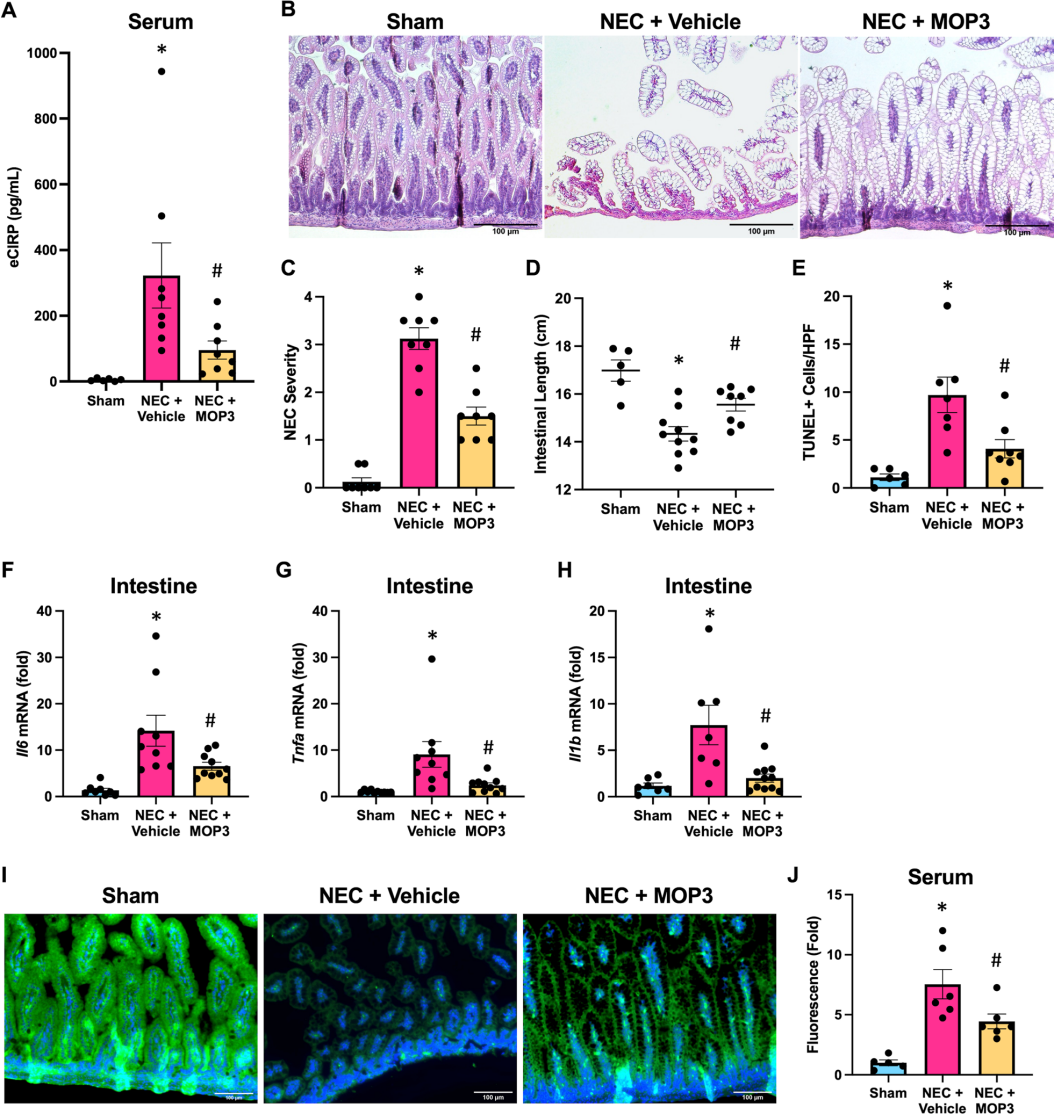
MOP3 protects against NEC pathogenesis, intestinal injury and inflammation, and barrier dysfunction. NEC was induced in WT pups, and pups were treated with MOP3 (20 μg/g, i.p.) or vehicle (volume equivalent) daily. Intestines were collected after 4 days of NEC. Age and litter-matched sham counterpart intestines were collected for comparison. **A** eCIRP levels detected in the serum from NEC mice. **B** Representative H&E images of sham and NEC intestines. Original magnification: 200x, Scale bar: 100 μm. **C** Quantification of NEC severity scoring of H&E intestinal sections. **D** Quantification of intestinal length from sham and NEC intestines. **E** Quantification of terminal deoxynucleotidyl transferase dUTP nick end labeling (TUNEL)–positive cells from intestinal sections. **F**–**H** Intestines were frozen and mRNA expression of IL-6, TNFα, and IL-1β was measured by PCR. **I** Sectioned intestines were stained by immunofluorescence for ZO-1 (green) and nuclear counterstained (blue). Original magnification: 200x, Scale bar: 100 μm. **J** Pups were administered FITC-dextran (FD4) by orogastric gavage 3-h prior to collection of blood and serum was analyzed for fluorescence intensity (expressed as fold-change from sham) as a measure of intestinal permeability. All experiments were performed 3 times, and all quantitative data were used for analysis (N = 6–11). Data are expressed as mean ± SEM and compared by One-way ANOVA and Tukey’s method. *p < 0.05 vs. sham, ^#^p < 0.05 vs. NEC + Vehicle

To validate the importance of eCIRP in NEC and determine its therapeutic potential, we sought to target eCIRP in a treatment model using MOP3, an eCIRP-targeted peptide. MOP3 treatment scavenged eCIRP as expected, as vehicle-treated NEC pups had elevated systemic levels of eCIRP, whereas MOP3 treatment significantly reduced these levels (Fig. 5A). In evaluating associated NEC severity, vehicle-treated NEC mice exhibited high severity of NEC, whereas MOP3-treated mice had reduced NEC severity (Fig. 5B, C). Consequently, the induction of NEC resulted in progressive loss of bowel length observed to a greater extent in vehicle-treated pups, whereas this was partially prevented in MOP3-treated mice (Fig. 5D). Intestines of MOP3-treated NEC pups had decreased numbers of TUNEL-positive apoptotic cells compared to vehicle-treated NEC pups (Fig. 5E). Furthermore, vehicle-treated NEC pup intestines demonstrated significantly increased IL-6, TNF-α, and IL-1β mRNA levels, whereas MOP3-treatment attenuated these levels in NEC (Fig. 5F–H).

To translate the sequelae of injury and inflammation, the intestinal barrier was again investigated through analysis of ZO-1. Induction of NEC with vehicle treatment resulted in depletion of ZO-1 in the intestinal epithelium, whereas the effect of ZO-1 depletion in NEC was blunted by MOP3 treatment (Fig. 5I). Using the fluorescent probe, FD4, to evaluate intestinal barrier function, vehicle-treated NEC pups demonstrated increased relative fluorescence intensity in the serum. Comparatively, MOP3 treatment significantly decreased sera fluorescence in NEC pups relative to vehicle, indicating preservation of the intestinal barrier by MOP3 (Fig. 5J). Together, these data support that MOP3 scavenges eCIRP and protects against intestinal injury, inflammation, and leaky gut permeability in NEC.

### MOP3 attenuates NEC-induced acute lung injury

**Fig. 6:**
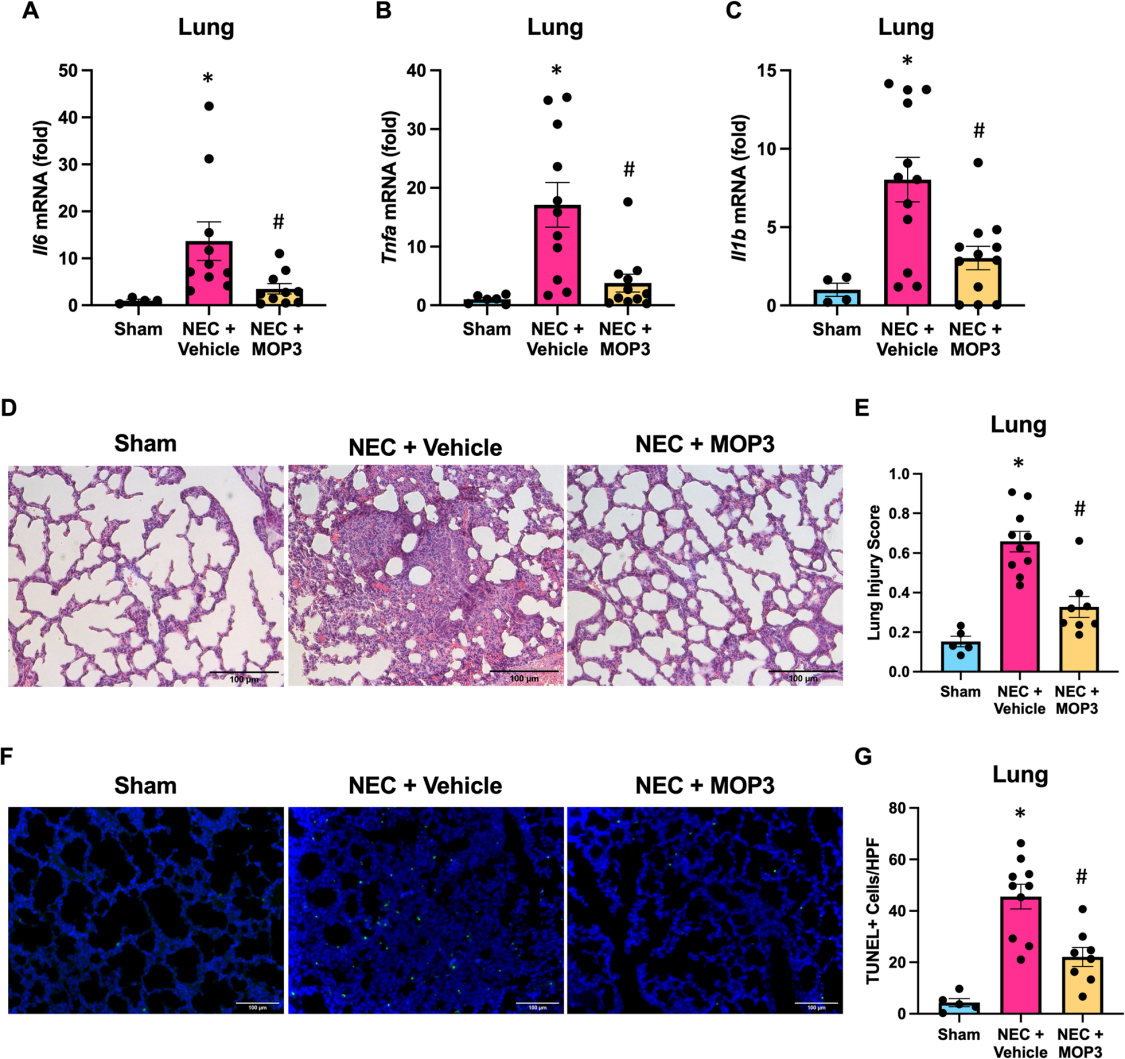
MOP3 attenuates NEC-induced acute lung injury. NEC was induced in WT pups, and pups were treated with MOP3 (20 μg/g, i.p.) or vehicle (volume equivalent) daily. Lungs were collected after 4 days of NEC. Age and litter-matched sham counterpart lungs were collected for comparison. **A**–**C** Whole lung tissue was frozen and mRNA expression of IL-6, TNFα, and IL-1β were measured by PCR. **D** Representative H&E images of NEC and sham lungs. Original magnification: 200x, Scale bar: 100 μm. **E** Quantification of lung injury of representative H&E lung sections by validated lung injury scoring. **F** Representative terminal deoxynucleotidyl transferase dUTP nick end labeling assay (TUNEL) staining (green) of sham and NEC lungs for apoptotic cells with nuclear counterstain (blue). Original magnification: 200x, Scale bar: 100 μm. **G** Quantification of TUNEL–positive cells from representative lung sections. All experiments were performed 3 times, and all quantitative data were used for analysis (N = 6–12). Data are expressed as mean ± SEM and compared by One-way ANOVA and Tukey’s method. *p < 0.05 vs. sham, ^#^p < 0.05 vs. NEC + Vehicle

To evaluate the impact of MOP3 on NEC-induced secondary organ injury, we evaluated the severity of lung inflammation and injury in this treatment model. Lung mRNA levels of IL-6, TNF-α, and IL-1β were increased in vehicle-treated NEC lungs, whereas these levels were significantly reduced in MOP3-treated counterpart lungs (Fig. 6A–C). There was significant histopathologic injury in vehicle-treated NEC pup lungs, whereas MOP3-treatment limited the degree of lung injury caused by NEC (Fig. 6D, E). Lastly, assessment of TUNEL-positive cells revealed a significant degree of lung cellular apoptosis in vehicle-treated NEC pups and attenuated lung cellular apoptosis in MOP3-treated NEC pups (Fig. 6F, G). Taken together, this data supports that MOP3 protects against exaggerated lung inflammation and injury induced by NEC.

### MOP3 reduces systemic inflammation and mortality in NEC

**Fig. 7:**
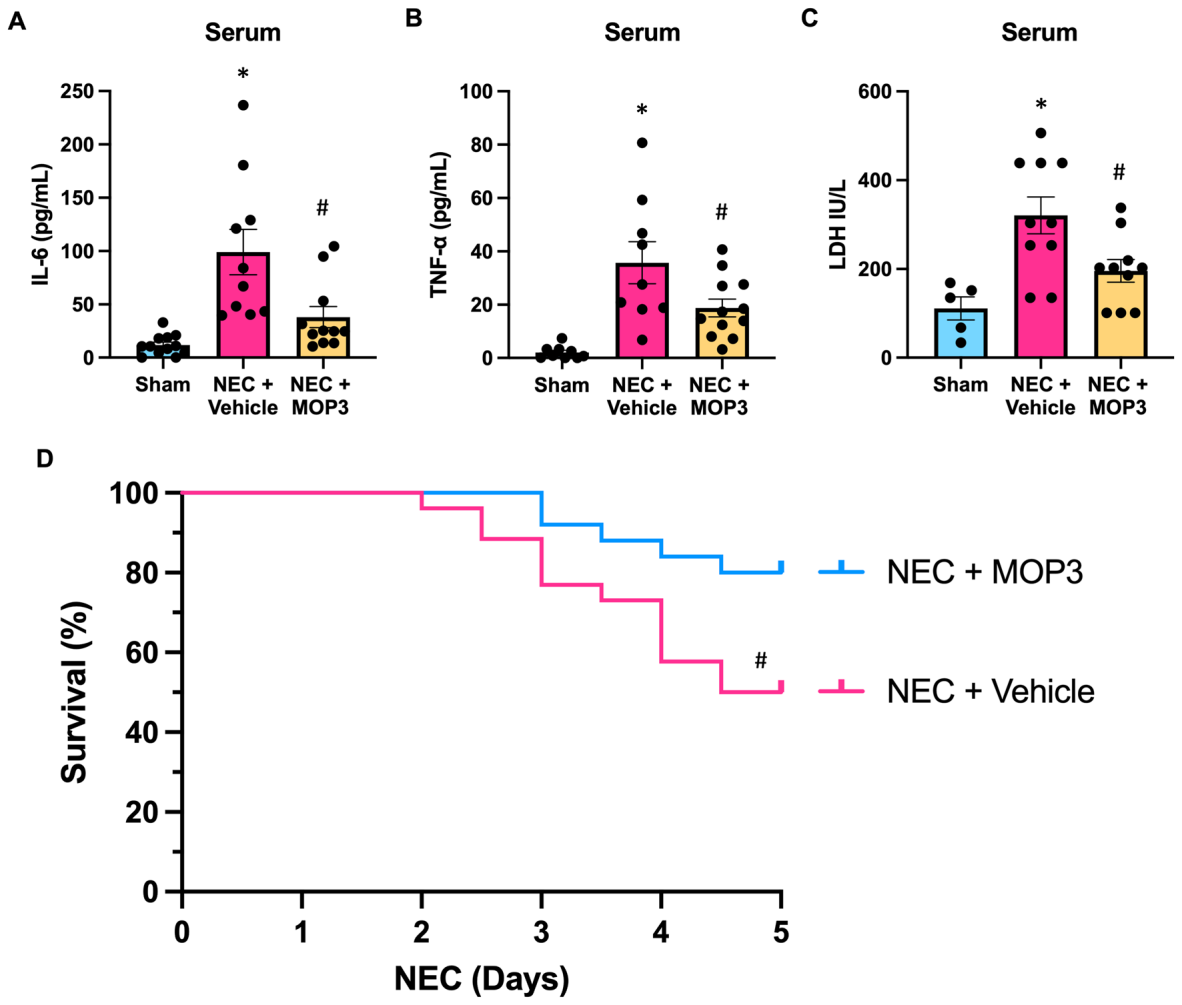
MOP3 reduces systemic inflammation and mortality in NEC. NEC was induced in WT pups, and pups were treated with MOP3 (20 μg/kg, i.p.) or vehicle (volume equivalent) daily. Blood was collected after 4 days of NEC. Age and litter-matched sham counterpart blood was collected for comparison. **A**–**B** Serum was separated and analyzed for systemic IL-6 and TNFα levels by ELISA. **C** Serum was analyzed for LDH levels by calorimetric assays. All experiments were performed 3 times, and all quantitative data were used for analysis (N = 6–12). Data are expressed as mean ± SEM and compared by One-way ANOVA and Tukey’s method. **D** Pups were subjected to NEC and treated with MOP3 (20 μg/kg, i.p.) or vehicle (volume equivalent) daily and monitored for differences in mortality in a 5-day survival study. Overall survival at 5-days was 80% for MOP3-treated pups compared to 50% for vehicle-treated pups (p = 0.024 by Kaplan Meier and log-rank test, N = 25–26/group). *p < 0.05 vs. sham, ^#^p < 0.05 vs. NEC + Vehicle; For survival curve: ^#^p vs. NEC + MOP3

Finally, to validate the beneficial impact imparted by MOP3 treatment in attenuating NEC, we sought to evaluate the overall systemic inflammatory profile. Vehicle-treated mice subjected to NEC had high systemic levels of IL-6, TNF-α, and LDH. Comparatively, MOP3 treatment significantly reduced these circulating inflammatory cytokines and marker of tissue injury in NEC (Fig. 7A–C). A critically important evaluative factor to determine therapeutic efficacy in NEC is to determine the impact on survival. At 5-days, there was a 50% mortality rate for vehicle-treated NEC pups. Incredibly, MOP3-treated pups exhibited improved survival, as the mortality was only 20% (Fig. 7D). Together, these data strongly support that targeting eCIRP with MOP3 is a promising therapeutic strategy to attenuate NEC, improve the inflammatory response, and prevent lethality in this disease.

## Discussion

Despite decades of NEC research, understanding of the pathophysiologic mechanisms contributing to NEC development has remained a challenge. Accordingly, due to the complexity as well as the insidious onset and rapid progression of this disease, effective treatments are still lacking. As the main barrier to the therapeutic breakthrough has been poor pathomechanistic understanding, we sought to elucidate undiscovered endogenous factors involved in NEC to drive therapeutic discovery. Thus, this work is the first to uncover that eCIRP exacerbates NEC pathogenesis and may be an effective therapeutic target. First, we have identified that neonates with NEC had elevated eCIRP levels in stool compared to health controls. In a preclinical model, genetic knockdown of CIRP significantly protected pups from NEC. Remarkably, our findings of the therapeutic benefits of MOP3—a peptide therapy designed to scavenge eCIRP—emphasizes the translational capability of these novel findings to treat NEC. Specifically, MOP3 treatment protected against intestinal villus injury, attenuated inflammation, reduced intestinal permeability, and prevented NEC-induced lung injury. MOP3 also significantly improved survival in NEC, further emphasizing the promise of this new therapeutic approach.

The importance of endogenous CAMPs in disease pathogenesis has long been recognized in the initiation of inflammatory cascades. Once released during states of cellular stress, eCIRP functions as a CAMP by recognizing pattern recognition receptors (PRRs) on surrounding immune cells and propagating inflammation (Aziz et al. 2019). Importantly, elevated plasma levels of eCIRP have been independently correlated with poor prognosis in human neonates with sepsis (Denning et al. 2020). This study is the first to implicate eCIRP levels as an early clinical diagnostic marker in NEC, for example through detection of elevated levels in the stool. Given that previous work has detected elevated eCIRP in the serum of septic neonates, serum eCIRP would likely also be elevated in NEC infants, given the systemic inflammation in NEC. Although not directly compared in this study, stool eCIRP may be a more specific marker for enteric inflammation compared to serum eCIRP (which may better capture systemic inflammation). Moreover, detection of DAMPs in the stool as a potential NEC biomarker has previously been explored. For example, HMGB1 elevation in the stool was found to be predictive of NEC risk (Vitali et al. 2021). However, levels of stool HMGB1 were not substantially different between infants with NEC stage II and III, which further highlights the need for greater research in the area to identify relevant biomarkers (Liu et al. 2022; Nofi et al. 2024c). Future work will investigate the specificity of stool eCIRP, and whether the degree of stool and serum eCIRP elevation may correlate with NEC severity and outcomes, which would enhance the efficacy of eCIRP as a diagnostic and prognostic marker. In addition, previous pre-clinical studies have demonstrated the deleterious role of recombinant CIRP and have supported the therapeutic benefit of targeting eCIRP, implicating it as an effective drug target in neonatal diseases (Denning et al. 2020, 2019; Nofi et al. 2024a, 2024b). For example, various eCIRP antagonists and competitive inhibitors decreased systemic inflammation, attenuated end-organ damage, and improved survival in preclinical neonatal sepsis models (Denning et al. 2020, 2019; Nofi et al. 2024b).

CAMPs, like eCIRP, are known to impart tissue damage through the activation of immune cells by binding PRRs and initiating downstream signaling pathways; however, this link has not yet been translated to NEC (Nofi et al. 2022). A critical PRR, toll-like receptor 4 (TLR4) has garnered tremendous attention for its role in predisposing the premature gut to NEC, and thus it is conceivable that TLR4 ligands should play a role in NEC onset (Niño et al. 2016). Activation of TLR4 by gram-negative bacteria colonizing the premature gut causes deleterious sequelae such as increased enterocyte apoptosis and augmented proinflammatory cytokine release (Lu et al. 2014). However, activation of TLR4 may occur not only through gut-colonizing exogenous bacteria, but also through eCIRP which is released at high levels during states of cellular stress. Thus, eCIRP may also contribute to the activation and propagation of inflammatory insult in NEC.

Novel pharmacological approaches have shown promise in targeting key pathways influencing the development and exacerbation of NEC, for example, as demonstrated by oligosaccharide TLR4 inhibitors (Neal et al. 2013; Wipf et al. 2015). Specifically, a TLR4 inhibitor reduced systemic inflammation in murine NEC and inhibited LPS-signaling in ex-vivo human NEC ileum (Neal et al. 2013). Although evidence supporting these novel therapies is encouraging, limited quality of human data precludes recommendation of clinical utilization (Pammi and Abrams 2015). Over the last decade, small synthetic peptides have emerged as a promising strategy in the arena of therapeutic development (Muttenthaler et al. 2021). Small oligopeptide therapeutics, such as MOP3, have exceptional potential to target protein–protein interactions involved in disease pathogenesis while maintaining high specificity and in vivo stability. Advances in pathomechanistic discovery and targeted treatments like MOP3 will fuel therapeutic momentum in the effort to cure NEC.

Additional anti-inflammatory factors have been identified to contribute to NEC pathogenesis and are important in therapeutic consideration (Hunter and Plaen 2014). Previously, evidence has supported the protective role of human breast milk in preventing NEC, which has been attributed to a multitude of anti-inflammatory glycoproteins, including MFG-E8 (Liu and Newburg 2013). The classic biologic function of MFG-E8 serves to decrease inflammation through clearance of apoptotic cells and plays an important role in modulating the immune response in NEC (Aziz et al. 2022, 2011; Chatterton et al. 2013). Although upregulated in the mammary gland during lactation, MFG-E8 is downregulated in acute inflammatory conditions (including NEC) (Wang 2014; Giuliani et al. 2016). Decreased levels of MFG-E8 have been associated with worse NEC severity in preterm neonates (Asaro et al. 2021). A new function of MFG-E8 has recently been identified whereby it clears eCIRP for degradation, thus conferring anti-inflammatory impacts (Nofi et al. 2024a). This further highlights the physiologic gap that is addressed by MOP3 treatment, whereby MOP3 not only scavenges high levels of pro-inflammatory eCIRP, but also replaces the roles served by its anti-inflammatory parent, MFG-E8. Thus, MOP3 may serve as an exciting new therapeutic option to treat NEC.

In evaluating the applicability MOP3 as a treatment in NEC, it is important to consider the translational limitations of murine models to recapitulate human NEC. Although key factors have been identified in the pathogenies of NEC, the exact sequence of biological events still remains poorly understood, making the development of animal models challenging (Singh et al. 2023). Nevertheless, this NEC model has been widely utilized, as it incorporates critical factors, such as formula enteral stress, the immunogenic component of gram-negative bacteria (LPS), and hypoxic stress (Lopez et al. 2023). Patterns of gene expression in intestinal immune cells as well as the microbiome resemble the human condition of NEC by similar animal models, further supporting its translational utility (Cho et al. 2020; Good et al. 2016; Warner et al. 2016). Although data on the microbiome was not directly collected in this study, further research should elucidate the impact of MOP3 on the changes in microbiota and the potential impact on NEC severity and outcomes. Critical biochemical and genetic pathways that have been identified to play a causative role in murine models have also been observed in human disease (including TLR4, IgA, EGF, and HMGB1) (Sodhi et al. 2010; Jilling et al. 2006; Werts et al. 2020; Hackam and Sodhi 2022). Thus, this widely established model allows reasonable investigation of this complex disease. Finally, in this treatment model, MOP3 was administered daily throughout the 4 days of NEC induction. It will be important for future studies to investigate whether this treatment may be beneficial as a rescue therapy, which will have clinical implications given the difficulty in diagnosing NEC and the potential to maintain MOP3’s efficacy as a delayed treatment.

## Conclusion

NEC remains the leading cause of morbidity and mortality for premature infants (Hackam and Sodhi 2022). Recent scientific advancements have highlighted major influencers of disease progression, including the activation of bacterial signaling receptors in premature intestines inciting an overwhelming inflammatory responses (Cho et al. 2020; Hackam and Sodhi 2022). Although previously unknown, eCIRP contributes to the pathophysiologic phenomena that drives NEC development. MOP3, an eCIRP-targeted small peptide therapeutic, attenuates NEC and prevents devastating disease sequelae, including death in murine models. Although challenges remain in treating this disease, a greater understanding of the pathomechanistic contributors to NEC development is essential to drive new therapeutic discovery, such as the development of MOP3, where efforts at the bench may drive clinical breakthroughs to cure NEC.

## Supplementary Information

Supplementary material 1

## Data Availability

The datasets used and/or analyzed during the current study are available from the corresponding author on reasonable request.

## Abbreviations

eCIRP: Extracellular cold-inducible RNA-binding protein
MFG-E8: Milk fat globule-epidermal growth factor-factor VIII
MOP3: MFG-E8-derived oligopeptide 3
DAMPs: Damage-associated molecular patterns
CAMPs: Chromatin-associated molecular patterns
LPS: Lipopolysaccharide
WT: Wild-type
AA: Amino acid
H&E: Hematoxylin and eosin
RFI: Relative fluorescence intensity
ZO-1: Zonula occludens-1
TUNEL: Terminal deoxynucleotidyl transferase dUTP nick end labeling assay
LDH: Lactate dehydrogenase
PRR: Pattern recognition receptor
TLR4: Toll-like receptor 4
