# Supplementary material for: An anti-eCIRP strategy for necrotizing enterocolitis
Source: Mol Med. 2024 Sep 20;30:156. doi: 10.1186/s10020-024-00935-3 (PMC11414128; doi:10.1186/s10020-024-00935-3)
Supplement: Supplementary file 1 — Supplementary material 1 [file 10020_2024_935_MOESM1_ESM.docx]

**Supplemental Table 1: PCR Primer Sequences**

| Primer | Forward Sequence | Reverse Sequence |
| --- | --- | --- |
| *β-actin* | 5’-CGTGAAAAGATGACCCAGATCA-3’ | 5’-TGGTACGACCAGAGGCATACAG-3’ |
| *Il6* | 5’-CCGGAGAGGAGACTTCACAG-3’ | 5’-CAGAATTGCCATTGCACAAC-3’ |
| *Tnfa* | 5’-AGACCCTCACACTCAGATCATCTTC-3' | 5’- TTGCTACGACGTGGGCTACA-3' |
| *Il1b* | 5’-CAGGATGAGGACATGAGCACC-3’ | 5’-CTCTGCAGACT-CAAACTCCAC-3’ |

**Supplemental Table 2: Clinical Characteristics of Neonates with Necrotizing Enterocolitis**

| **Case** | **Gestational Age (weeks)** | **Age at diagnosis (days)** | **Clinical Condition** |
| --- | --- | --- | --- |
| **1** | 24 | 102 | Presented with abdominal distension and exhibited distended loops of bowel on abdominal Xray. Diagnosed with NEC. |
| **2** | 25 | 20 | Presented with abdominal distension and exhibited pneumatosis on abdominal Xray. Diagnosed with NEC. |
| **3** | 25 | 21 | Presented with bloody stools and exhibited bowel wall edema on abdominal Xray. Diagnosed with NEC. |
| **4** | 25 | 49 | Presented with abdominal distension with absent bowel sounds and hemodynamic instability. Diagnosed with NEC. |
| **5** | 27 | 14 | Presented with abdominal distention while feeding fortified feeds. Diagnosed with intestinal perforation versus NEC. |
